# Rotational Stability of a New Posterior Chamber Toric Phakic Intraocular Lens

**DOI:** 10.1155/2020/1624632

**Published:** 2020-04-09

**Authors:** Dilek Yaşa, Bülent Köse, Alper Ağca

**Affiliations:** ^1^Beyoğlu Eye Training and Research Hospital, Bereketzade Mah, No. 2, Beyoglu, Istanbul, Turkey; ^2^Aritmi Osmangazi Hospital, Ulu Mah, Ulubatlı Hasan Bulvarı, No. 48-62, Bursa, Turkey

## Abstract

**Purpose:**

To evaluate the refractive results and rotational stability of Eyecryl toric phakic intraocular lens (pIOL).

**Methods:**

In this retrospective single-surgeon case series, manifest refraction, visual acuities, endothelial cell density (ECD), and pIOL rotation were evaluated over 6 months.

**Results:**

43 eyes from 23 patients were included. At 6 months, the SE was within ±0.50**  ***D* of emmetropia in 30 (70%) eyes and within ±1.00**  ***D* of emmetropia in 39 (91%) eyes. The efficacy and safety indices were 1.25 ± 0.38 and 1.41 ± 0.34, respectively. Mean ECD was 2719 ± 296 cells/mm^2^ at the preoperative visit and 2779.42 ± 422 cells/mm^2^ at the 6-month visit (*p* > 0.05). The mean value of absolute axis orientation error was 4.95 ± 5.28°. Mean absolute change in axis orientation between visits was less than 3° for all visit intervals. Ninety percent or more of lenses were found to rotate 5° or less between all visit intervals. None of the patients experienced a vision-threatening complication, and no patient required secondary IOL repositioning.

**Conclusion:**

The pIOL appears to effectively reduce subjective manifest astigmatism and provide good visual acuity. Its position was found to be stable throughout the follow-up.

## 1. Introduction

Phakic intraocular lens (pIOL) implantation was first proposed by Strambelli in 1954, and it may correct a high amount of myopia and astigmatism [1]. Some models of pIOLs are considered safe and effective [2–4], while others have been taken off the market because of complications [5]. Only one posterior chamber pIOL (PC-pIOL) (Visian Implantable Collamer Lens; Staar Surgical Company, Monrovia, CA) has a long history of follow-up and meets the efficacy and safety criteria of the US Food and Drug Administration (FDA) [2]. One PC-pIOL (phakic refractive lens, Zeiss Meditec, Germany) was associated with zonular dehiscence and have been taken off the market despite satisfactory refractive outcomes [6–8]. There are only a few short-term studies with other PC-pIOLs [9, 10].

Rotational orientation and the stability of a toric IOL are crucial for a satisfactory refractive outcome because a rotational error of just 5° results in a 17% loss of cylindrical power. Eyecryl Toric pIOL (Biotech Vision Care, Ahmedabad, India) is a new toric PC-pIOL and, to the best of our knowledge, there are no published studies that describe its rotational stability.

The objective of this study was to evaluate the rotational stability and the early refractive results and safety of Eyecryl Toric pIOL.

## 2. Patients and Methods

The study adhered to the tenets of the Declaration of Helsinki. Prior approval from the Ethics Committee of Taksim Training and Research Hospital was obtained. Patients who had Eyecryl Toric pIOL implantation in our clinic were included in this study. The patients who had a follow-up of less than six months were excluded.

Uncorrected (UDVA) and corrected distance visual acuities (CDVA) were measured with an LCD chart and a digital phoropter. Corneal topography was performed with a Sirius (Costruzione Strumenti Oftalmici, Italy) topography system. Preoperative anterior chamber depth and postoperative pIOL vault were measured with a Visante OCT (Carl Zeiss AG, Germany). A CEM 530 (NIDEK, Japan) specular microscope was used to measure central endothelial cell density (ECD). Optical biometry was performed preoperatively with an AL-Scan (NIDEK, Japan). Postoperative dilated anterior segment photography was performed in follow-up visits.

**Figure 1:**
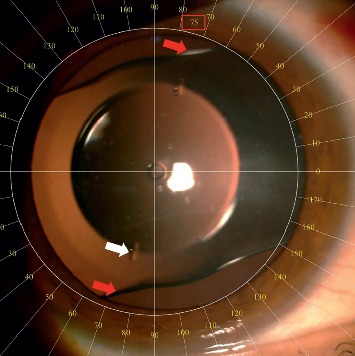
Eyecryl toric phakic IOL implanted in the posterior chamber. White arrow shows axis marks on the IOL. Red arrows and the red rectangle indicate the measurement result.

The ANSI standard toric IOLs (Z80.30-2010) require that 90% or more of the lenses rotate 5° or less between visits that are approximately 3 months apart. Thus, lens positions on 1-day, 3-month, and 6-month visits were compared to evaluate rotational stability. Goniotrans, a free standalone application (available at https://www.goniotrans.com/), was used to measure the angular position of the pIOL during postoperative visits (Figure 1). A free standalone application was used for vector analysis [11].

### 2.1. Phakic Intraocular Lens

The Eyecryl Toric pIOL is a plate haptic PC-pIOL made up of hydrophylic acrylic/collagen-based biocompatible polymer material, and its design is similar to Visian ICL at the first look (Figure 1). The IOL material is a copolymer of hydrophilic acrylic material and bovine collagen. It has an aspherical optic (zero aberration) with a central hole (320 *µ*m). The IOL is available in three overall lengths.

### 2.2. Surgical Procedure

Approximately 10 minutes before the surgery, reference marks were placed at 0- and 180-degrees at the slit lamp by turning slit illumination horizontally and using it as a reference. At the beginning of the surgery, before entering the anterior chamber, a Mendez ring was positioned on the eye so that the 0- and 180-degree marks on the ring matched the previously placed reference marks. The desired implantation axis was than marked with a marking pen using the Mendez ring as a reference.

A paracentesis incision was performed, and adrenalin was injected into the anterior chamber before loading the IOL into the cartridge/injector system. After the IOL was loaded into the cartridge/injector system, a cohesive OVD (Provisc; Alcon, Ft Worth, TX) was injected into the anterior chamber. A temporal 2.75 mm clear corneal incision was performed, and the pIOL was implanted into the anterior chamber (over the iris) through the incision. The haptics of the pIOL were then gently positioned in the sulcus one by one, and the pIOL was rotated so that the marks on the IOL were parallel to the imaginary line (between marks) indicating the implantation axis. The OVD was simply washed out of the anterior chamber, and incisions were hydrated with a balanced salt solution.

### 2.3. Statistical Analysis

Statistical analysis was performed using SPSS for Windows (version 21.0, IBM Corp.). Normal distribution was evaluated using the Shapiro–Wilk test. Endothelial cell counts, SE manifest refraction, and pIOL axis (during follow-up) were analyzed with repeated measures analysis of variance (ANOVA).

## 3. Results

Forty-three eyes of 23 patients were included in the study. Ten (43%) patients (17 (40%) eyes) were male, and 13 (57%) patients (26 (60%) eyes) were female. The mean age of the patients was 32 ± 8 years. Preoperative characteristics of the eyes are listed in Table 1.

**Figure 2:**
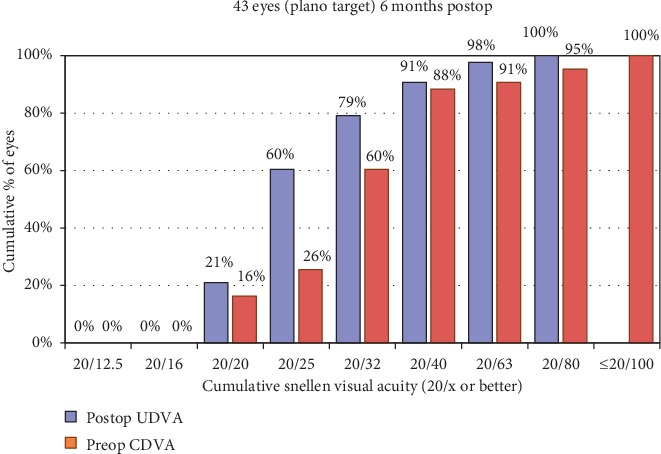
Uncorrected distance visual acuity.

The cumulative percentages of postoperative UDVA and preoperative CDVA are reported in Figure 2. The mean preoperative CDVA was 0.23 ± 0.16 logMAR. The efficacy index (postoperative UDVA/preoperative CDVA) at the 6-month time point was 1.25 ± 0.38.

**Figure 3:**
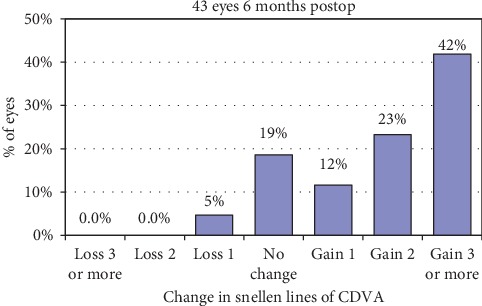
Change in corrected distance visual acuity.

The mean postoperative CDVA was 0.09 ± 0.12 logMAR, and the mean safety index was 1.41 ± 0.34. The changes in CDVA lines are reported in Figure 3. No patient lost 2 or more lines of CDVA, and 65% of the eyes gained 2 or more lines of CDVA. Mean ECD was 2719 ± 296 cells/mm^2^ during the preoperative visit and 2779 ± 422 cells/mm^2^ during the 6-month visit (*p*=0.260, paired samples *t*-test). None of the patients experienced a vision-threatening complication, and no patient required a secondary IOL repositioning.

**Figure 4:**
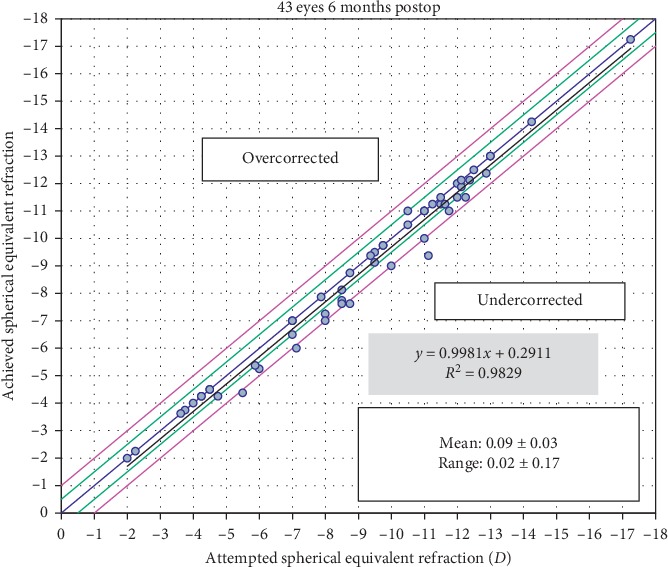
Attempted versus achieved refraction.

**Figure 5:**
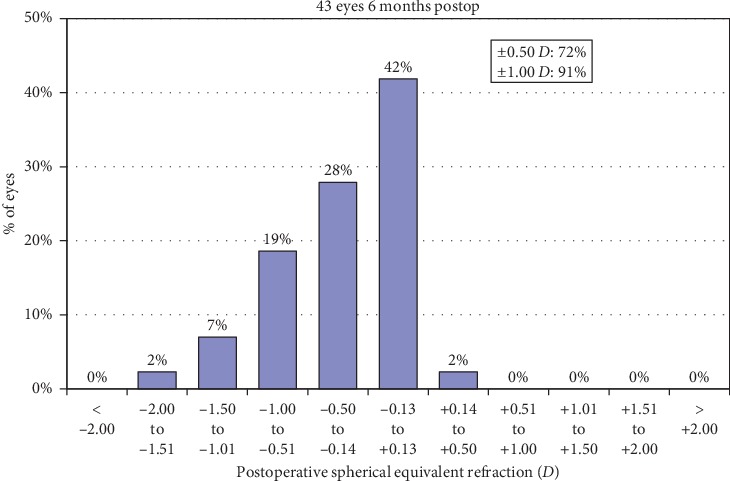
Refractive accuracy (spherical equivalent refraction).

**Figure 6:**
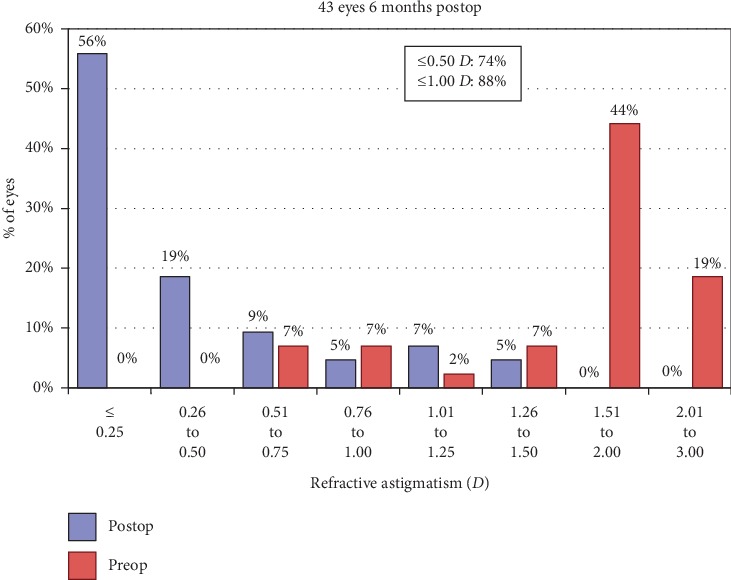
Refractive accuracy (astigmatism).

**Figure 7:**
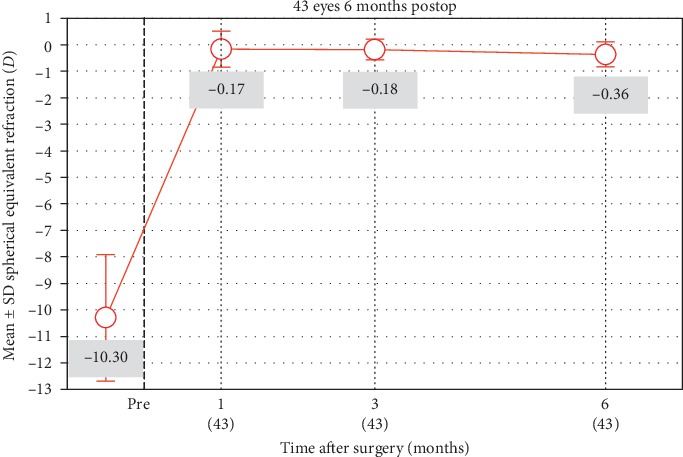
Stability of spherical equivalent refraction.

A scatterplot of the attempted versus achieved corrections is shown in Figure 4. 6 months after surgery, 30 (70%) and 39 (91%) of the eyes were within ±0.50**  ***D* and ±1.00**  ***D* of the attempted correction, respectively (Figure 5). Postoperative astigmatism was 0.50**  ***D* or less in 32 (74%) eyes and 1.00**  ***D* or less in 38 (88%) eyes (Figure 6). SE refraction was −0.17 ± 0.68 and −0.36 ± 0.47**  ***D* during the 1- and 6-month visits, respectively (Figure 7). Although the difference was small, it was statistically significant (*p*=0.014, paired samples *t*-test).

In 38 (88%) of the eyes, the phakic IOL was within ±5.00° of the intended meridian on postoperative day 1. The mean value of the absolute axis orientation error was 4.95 ± 5.28°. The mean values of target-induced astigmatism, surgically induced astigmatism, and difference vector were 1.12**  ***D* at 90°, 1.03**  ***D* at 90°, and 0.09**  ***D* at 95°, respectively. The arithmetic and geometric means of correction index were 0.96 and 0.95, respectively.

### 3.1. Rotational Stability

Table 2 shows the absolute change in axis orientation between visits. Ninety percent or more of lenses were found to rotate less than 5⁰ between all visits, and the mean change in axis orientation was found to be <3° for all visit intervals.

## 4. Discussion

Efficacy of Eyecryl Toric pIOL has not been evaluated before. However, Yasa et al. [10] reported that 62% of the eyes were within ±0.50 *D* of the attempted correction after implanting a nontoric version of the same pIOL. In agreement with that study, we found that the SE was within ±0.50**  ***D* of the intended correction in 70% of the eyes and within ±1.00**  ***D* in 91% of the eyes. Manifest refraction is an important variable in vergence formulas. However, performing subjective manifest refraction examination is difficult if the patient has a high refractive error and low visual acuity. Thus, predictability after pIOL implantation depends on the preoperative patient characteristics. Accordingly, it is possible that the percentage of eyes within ±0.50 *D* would be higher if SE was lower and the DCVA was better in our patient group. For example, Alfonso et al. [12] reported outcomes after the implantation of ICL in 35 eyes with a mean SE of −7.03 ± 4.07 D. At 12 months, 97% of eyes were within ±0.50**  ***D* of the target. Hashemian et al. [13] evaluated the toric ICL in 64 eyes with a mean SE of −11.67 ± 3.99**  ***D* (from −3.0 to −21.0**  ***D*). They reported that 40.5% and 34.4% of patients were within ±0.5**  ***D* of the attempted correction at 6- and 12-month postoperative follow-ups, respectively. In line with previous studies with pIOLs, we found that the efficacy and safety index were both higher than 1.0 [10, 14].

We found that there was a small, but statistically significant increase in the mean SE refraction during the 6-month visit compared to 1 month postoperative. This was an expected finding because phakic IOLs are usually preferred in young patients with very high myopia. Because of the progressive nature of myopia in most of these patients, the efficacy of the procedure decreases with time [15]. Corneal diseases such as keratoconus or development of cataracts may also induce progression of myopia [16]. However preoperative corneal topography was normal in all eyes and cataracts did not developed in any eye. In our opinion, the increase in the mean SE refraction is not surprising and results from an increase in the mean AL of the eyes. However, it was not possible to evaluate postoperative AL due to the retrospective nature of the study.

Rotational stability of a toric IOL is very important for satisfactory refractive correction. Thus, we evaluated 1-day, 3-month, and 6-month visits in this study and compared every visit interval. We found that ninety percent or more of lenses rotate 5° or less between all visits, and mean change in axis orientation was <3° for all visit intervals (Table 2). In this manner, the results in our study group exceeds the ANSI standards. Our measurements of rotational stability are similar to other toric IOLs [13, 17, 18]. In a prospective clinical trial, Hashemian et al. [13] found that there was ≥10, ≥15, and ≥20 degrees of rotation in 8.3%, 2.8%, and 2.8% of the toric ICLs, respectively. Hyun J et al. [17] found that 79.2% of eyes with V4c toric ICL and 70.8% of eyes with V4 toric ICL had a rotational stability under 5°, until the last follow-up. They also reported that 8.3% of eyes with V4 toric ICL rotated >10° and determined that there was no statistically significant difference between the two designs. In this study, ≥10° rotation was seen in 3 (6.9%) eyes. In these eyes, the phakic IOLs rotated ≥10° at 1-month when compared to the 1-day visit and they were stable (rotated ≤5°) during the rest of the follow-up. In all three eyes, the pIOL vault at 1 month was <250 microns. Although, the majority of eyes with a low vault maintains clear lenses, a low vault is a risk factor for early cataract formation [19]. Thus, our patients were informed about the increased incidence of cataracts. A pIOL exchange operation was offered for increased UDVA, and potential complications of this second surgery were discussed with the patients. However, all three patients were already satisfied with the improved visual acuity and opted to be under close follow-up instead of a pIOL exchange. Thus far, no complications developed in these three patients during follow-ups. In this study, the size was based on WTW measurements, following the manufacturer's recommendations. More than 10° rotation in three pIOLs in this study during the early postoperative period may be related to improper sizing. Unfortunately, one of the main problems with posterior phakic IOL implantation is choosing the correct size IOL for the ciliary sulcus. Zeng et al. [20] reported that 2.6% of the ICLs were replaced because of too high or too low vaults. We would like to highlight that direct measurement of the horizontal STS diameter was found not to improve postoperative results [21]. Also, one should keep in mind that even the correct horizontal sulcus diameter would not be enough to describe the correct shape of the sulcus in a given patient because the ciliary sulcus is not circular. To complicate the issue further, there are other variables that are not measurable such as the posterior compression by the iris or the ciliary sulcus structure [22].

The major limitation of this study is its retrospective nature. A 6-month follow-up is sufficient to evaluate refractive results and rotational stability, which were the main outcome measures in this study, but we would like to highlight that it is not long enough to the study safety. Complications such as pigment dispersion, endothelial cell loss, cystoid macular edema, and glaucoma are reported with phakic IOLs and other intraocular lenses which are not implanted in the capsular bag [5, 6, 23, 24]. However, no patient in this study developed a cataract, glaucoma, significant endothelial cell loss, or any other complication. Also, the mean ECD during the postoperative 6-month visit was not significantly different from preoperative ECD. Other studies have reported 1-year [10] and 2-year [25] results after a nontoric version (which was not used in this study), and the low complication rates in these studies are in line with the results reported here.

In conclusion, we have found that the refractive results and rotational stability of Eyecryl toric phakic IOL are similar to other toric PC-pIOL models. Prospective controlled studies are needed to evaluate cataract formation rates.

## Figures and Tables

**Table 1:** Preoperative characteristics.

	Mean ± SD	Range
SE (D)	−10.30 ± 2.38	−5.50 to −17.25
Astigmatism (D)	2.11 ± 1.04	0.75 to 5.50
Mean Sim-K (D)	44.61 ± 1.82	39.04 to 48.22
Corneal thickness (*µ*m)	526 ± 36	429 to 595
White-to-white (mm)	11.73 ± 0.29	11.18 to 12.09
ACD (mm)	3.23 ± 0.26	3.00 to 4.11
Axial length (mm)	26.89 ± 1.07	24.28 to 29.61

ACD: anterior chamber depth from the endothelium; white-to-white: horizontally visible iris diameter; Sim-K: mean simulated keratometry; SE: spherical equivalent of manifest refraction.

**Table 2:** Absolute change in axis orientation between visits.

	Absolute rotation	Lens rotation ≤5°	Lens rotation ≤10°
Visit range	(mean ± SD)	*n* (%)	*n* (%)
1 day to 3 months	2.33 ± 3.80	40 (93.0)	40^*∗*^ (93.0)
3 to 6 months	1.35 ± 1.67	41 (95.3)	43 (100.0)
1 day to 6 months	2.95 ± 3.75	40 (93.0)	40^*∗*^ (93.0)

*n*: number of eyes; SD: standard deviation. ^*∗*^In three eyes, there was >10° lens rotation between 1-day and 1-month visits. In these eyes, the pIOLs rotated ≤5° during the rest of the follow-up.

## Data Availability

The data that support the findings of this study are available from the corresponding author (DY) upon reasonable request.
